# Maternal Impressions

**Published:** 1863-10

**Authors:** John S. Beale

**Affiliations:** Paddington-green


### MATERNAL IMPRESSIONS.

To the Editor of The Lancet:

Sir:—The following case may perhaps be deemed worthy of a place in your pages:

Mrs. N——, aged twenty-four years, primipara, a woman of delicate build, and highly nervous temperament, suffered a great deal during her pregnancy with morning sickness, faintings, and great despondency of spirits. About ten weeks previous to her delivery she had a few scattered spots of "herps" on the front of the chest, which disappeared under ordinary treatment, when some kind, good-natured, knowing old woman informed her it was the "small-pox, and that without doubt her child would suffer from the same disease." The bare notion of this preyed very much upon her mind, and her husband and myself both failed in driving the absorbing notion from her brain.

On June 22d she sent for me, having been in labor some four hours. On rupturing the membranes, a most unusual quantity of amniotic fluid escaped, coming away in gushes with the commencement of each pain. On the child being born, I noticed it had been dead for several days, the head, face, and whole surface of the body being covered at about three-quarter inch intervals with pustules exactly resembling in size, form, and appearance the small-pox vesicles at maturity. The depression in the centre was plainly marked. When the topmost cuticle was detached, there was no fluid of any sort underneath. The mother's first remark was, "Is the child marked?" she fully believed it would be so. We well know how mysterious are the freaks of nature in cases of this description. Still I think a careful complication of numerous cases would tend eventually to throw more light on the matter.

The following cases have occurred in my practice:

1—A child born with one eye of a light blue color (right eye); the other a dark hazel. Mother says she had seen a child with similar eyes sitting on a door-step in Lisson-grove.

2—Child born with mouth and upper and lower extremities resembling those of a dog. Mother states that she was worried and torn by a dog whilst she was in the seventh month of gestation.

3—Child born with left eye blackened as from a block. The mother stated that her husband came home irritated, and struck her (eight hours previous to her confinement) on the corresponding part of her face.

4—A child born with four little fins or stumps for upper and lower extremities. The mother had been frightened by seeing a man maimed in his lower extremities, who used to traverse the streets on a board with wheels.

5—Child born ten nights after display of fire-works in commemoration of Crimean war. Child's feet were covered with bladders of serum, similar to those arising from scald or burn. The mother was alarmed by the descent of a stick of a discharged fire-rocket, which struck the roof close by the place where she was standing.

I remain, Sir, your obedient servant,

John S. Beale, M. R. C. S. L.

Paddington-green, June, 1863.

